# Dihydroartemisinin-piperaquine or sulphadoxine-pyrimethamine for the chemoprevention of malaria in children with sickle cell anaemia in eastern and southern Africa (CHEMCHA): a protocol for a multi-centre, two-arm, double-blind, randomised, placebo-controlled superiority trial

**DOI:** 10.1186/s13063-023-07274-4

**Published:** 2023-04-05

**Authors:** Thandile Nkosi-Gondwe, Bjarne Robberstad, Robert Opoka, Dennis Kalibbala, Joseph Rujumba, Lufina Tsirizani Galileya, Pamela Akun, Winnie Nambatya, John Ssenkusu, Feiko TerKuile, Kamija Phiri, Richard Idro

**Affiliations:** 1 Training and Research Unit of Excellence, Blantyre, Malawi; 2 University of Bergen, Bergen, Norway; 3 Makerere University College of Health Sciences, Kampala, Uganda; 4 Global Health Uganda, Kampala, Uganda; 5 Liverpool School of Tropical Medicine, Liverpool, UK

**Keywords:** Malaria, Sickle cell anaemia, Chemoprevention, Mortality, Dihydroartemisinin-piperaquine, Sulphadoxine-pyrimethamine

## Abstract

**Background:**

An estimated 300,000 babies are born with sickle cell anaemia (SCA) annually. Affected children have chronic ill health and suffer premature death. Febrile illnesses such as malaria commonly precipitate acute crises in children with SCA. Thus, chemoprophylaxis for malaria is an important preventive strategy, but current regimes are either sub-optimally effective (e.g. monthly sulphadoxine-pyrimethamine, SP) or difficult to adhere to (e.g. daily proguanil). We propose dihydroartemisinin-piperaquine (DP) as the agent with the most potential to be used across Africa.

**Methods:**

This will be a randomised, double-blind, parallel-group superiority trial of weekly single-day courses of DP compared to monthly single-day courses of SP in children with SCA. The study will be conducted in eastern (Uganda) and southern (Malawi) Africa using randomisation stratified by body weight and study centre. Participants will be randomised using an allocation of 1:1 to DP or SP. We will investigate the efficacy, safety, acceptability and uptake and cost-effectiveness of malaria chemoprevention with weekly courses of DP vs monthly SP in 548 to 824 children with SCA followed up for 12–18 months. We will also assess toxicity from cumulative DP dosing and the development of resistance. Participant recruitment commenced on 30 April 2021; follow-up is ongoing.

**Discussion:**

At the end of this study, findings will be used to inform regional health policy. This manuscript is prepared from protocol version 2.1 dated 1 January 2022.

**Trial registration:**

The trial was registered at ClinicalTrials.gov, NCT04844099. Registered on 08 April 2021.

## Administrative information

Note: the numbers in curly brackets in this protocol refer to the SPIRIT checklist item numbers. The order of the items has been modified to group similar items (see http://www.equator-network.org/reporting-guidelines/spirit-2013-statement-defining-standard-protocol-items-for-clinical-trials/).

**Table:** 

Title {1}	Dihydroartemisinin-Piperaquine or Sulphadoxine-Pyrimethamine for the Chemoprevention of Malaria in Children with Sickle Cell Anaemia in eastern and southern Africa (CHEMCHA): A protocol for a multi-centre, two-arm, double-blind, randomized, placebo-controlled superiority trial
Trial registration {2a and 2b}.	NCT04844099 [ClinicalTrials.gov] [registered on 08 April 2021]
Protocol version {3}	1^st^ January 2022, v2.1
Funding {4}	This study is funded by the Research Council of Norway, Global Health and Vaccination Research (GLOBVAC) programme.
Author details {5a}	Thandile Nkosi—Gondwe: Training and Research Unit of Excellence, 1 Kufa Road, Blantyre, Malawi. Bjarne Robberstad: Robert Opoka: Makerere University College of Health Sciences, P.O Box 7072, Kampala, Uganda Dennis Kalibbala: Global Health Uganda, P.O BOX 33842. Kampala, Uganda Joseph Rujumba: Makerere University College of Health Sciences, P.O Box 7072, Kampala, Uganda Lufina Tsirizani Galileya: Training and Research Unit of Excellence, 1 Kufa Road, Blantyre, Malawi. Pamela Akun: Makerere University College of Health Sciences, P.O Box 7072, Kampala, Uganda Winnie Nambatya: Makerere University College of Health Sciences, P.O Box 7072, Kampala, Uganda John Ssenkusu: Makerere University College of Health Sciences, P.O Box 7072, Kampala, Uganda Feiko Ter Kuile: Liverpool School of Tropical Medicine, Pembroke Place Liverpool L3 5QA UK Kamija Phiri: Training and Research Unit of Excellence, 1 Kufa Road, Blantyre, Malawi. R. Idro: Makerere University College of Health Sciences, P.O Box 7072, Kampala, Uganda
Name and contact information for the trial sponsor {5b}	Helen Wong, Programme Officer, Department of Clinical Sciences (Room M220) Liverpool School of Tropical Medicine Pembroke Place Liverpool, L3 5QA, UK Telephone: + 44(0)151 705 3346 E-mail: Helen.Wong@lstmed.ac.uk
Role of sponsor {5c}	The study sponsor and funder had no role in study design and other than study oversight, will play no role in the collection, management, analysis, and interpretation of data; writing of the report; and the decision to submit the report for publication.

## Introduction

### Background and rationale {6a}

Sickle cell anaemia (SCA) is one of the most common inherited disorders in the world [1]. Patients suffer chronic morbidity and early mortality mainly due to severe complications related to hypoxia. It is estimated that SCA causes 5–16% of all under-5 mortality in sub-Saharan Africa. Although significant gains have been made, malaria too remains a major cause of morbidity and mortality in African children [2]. Children with the heterozygous sickle cell trait (HbAS) are strongly protected against severe malaria [3], and this is thought to be the main reason why the HbS gene has been retained in African populations. Children with the homozygous SCA also appear to have a lower risk of malaria compared to those without but may suffer much higher mortality if hospitalised with malaria [4]. The reasons for the increased risk may partly be related to worsened anaemia, sickle cell crises and bacteraemia that can occur with malaria. Thus, in many African countries, malaria chemoprevention is prescribed for the patients. However, due to high levels of parasite resistance, regimes such as monthly sulphadoxine-pyrimethamine (SP) that were recommended decades ago may now be sub-optimally effective. Despite this, guidelines in Uganda and Malawi, among others, still recommend monthly SP [5]. Daily regimes, e.g. daily proguanil, are recommended in other countries such as Kenya but are difficult to adhere to. In West Africa, the recommendations are even more unclear. For example, in Nigeria, SCA guidelines recommend daily proguanil, but the National Malaria Control Program recommends against this as proguanil may be ineffective. Instead, a policy of no chemoprevention but a test and treat strategy is recommended. Moreover, compared to eastern and southern Africa, a high burden of infections in West Africa is due to vivax malaria. The striking differences in aetiology and approach, coupled with the compelling but limited evidence that malaria is a major cause of mortality in SCA, provide a strong reason for multi-country studies.

Artemisinin-containing combination therapies (ACTs) such as DP are now the standard for the treatment of *Plasmodium falciparum* malaria in both children and adults [6]. A systematic review of the efficacy and safety of ACTs showed that DP is very effective and provides a long duration of post-treatment prophylaxis similar to mefloquine and a longer duration than amodiaquine or lumefantrine-based combinations [6, 7]. Because of its long half-life, piperaquine has great potential for use as the ACT of choice for intermittent treatment and chemoprophylaxis, as was shown in intermittent preventive treatment (IPT) studies in children [8]. We propose dihydroartemisinin-piperaquine as the chemopreventive agent of choice for children with SCA on the continent.

A trial with monthly DP used as IPT in Thai adults showed it to be well-tolerated, safe and highly effective [9]. The most important determinant of protective efficacy was the trough plasma concentration of piperaquine, and this was determined by the dosing frequency. The study suggested that for effective prevention of malaria, DP should at least be given monthly to achieve steady-state concentrations above the minimum inhibitory concentrations and sustained prophylactic levels. Piperaquine is currently only available in the fixed-dose combination with dihydroartemisinin (DHA) as DP. The DHA component, eliminated within a few hours, is not expected to contribute significantly to chemoprevention effects, yet may provide a degree of protection against the development of piperaquine resistance in the population.

Side effects of DP in adults include transient drops in haemoglobin by day 7 (seen with all ACT), headache, weakness, and fever. Its main safety concerns relate to its dose-dependent QT prolongation seen in clinical trials, including our post-discharge chemoprevention of malaria in transfused children (PMC) study [10]. Nevertheless, the side effects of DP are mild and similar to that seen with other anti-malarial drugs [11]. There is also no indication from the data signalling that it is associated with clinically significant arrhythmia [12].

On the other hand, SP is one of the most successful drugs for malaria prophylaxis [13]. In Uganda, monthly SP was more efficacious than weekly chloroquine in malaria prophylaxis in children with SCA [14]. However, this combination has increasingly become ineffective in Africa [15]. Resistance is mediated through mutations at the genes encoding *P. falciparum* dihydrofolate reductase (Pfdhfr) and dihydropteroate synthase (Pfdhps). In eastern and southern Africa, more than 90% of parasites harbour up to three mutations in the dihydrofolate reductase [dhfr] gene and two mutations in the dihydropteroate synthase [dhps] gene [16, 17]. This high-level burden of resistance genes may mean ineffective malaria chemoprevention. In this trial, we aim to determine the efficacy and safety of malaria chemoprevention with weekly single-day courses of DP compared to monthly single courses of SP (the standard of care).

## Objectives {7}

### Primary objective

The study objective is to determine the efficacy and safety of malaria chemoprevention with weekly single-day courses of DP compared to monthly single courses of SP in children with SCA in eastern and southern Africa.

### Secondary objectives

1. Assess the feasibility and stakeholder perceptions on the uptake (acceptability) and the potential for future roll-out of weekly DP vs monthly SP
2. Determine the safety of cumulative dosing of DP, especially on cardiac function
3. Monitor the development of malaria parasite resistance to DP in clinical isolates over time
4. Assess patients’ health-related quality of life, cost-effectiveness, equity and economic implications of using weekly courses of DP vs monthly courses of SP as chemoprevention in SCA
5. Conduct policy advocacy to engage key stakeholders on policy decisions on using weekly courses of DP or monthly courses of SP for the chemoprevention of malaria in SCA

## Trial design {8}

This is a multi-centre, parallel-group, two-arm, double-blind, individually randomised superiority trial with a 1:1 allocation ratio comparing the efficacy, safety, acceptability, uptake and cost-effectiveness of malaria chemoprevention with weekly courses of DP or monthly SP in children with SCA in eastern (Uganda) and southern (Malawi) Africa. Randomisation to either weekly DP + monthly SP-*placebo* or monthly SP + weekly DP-*placebo* is by varying block sizes and stratified by study site and weight. The primary outcome is the incidence of clinical malaria defined as a history of fever in the preceding 48 h or documented axillary temperature ≥ 37.5 °C plus *Plasmodium falciparum* parasites on microscopic examination of a blood smear.

## Methods: participants, interventions and outcomes

### Study setting {9}

This study is being conducted in Uganda and Malawi. These two countries have moderate to intense malaria transmission. The study sites in Uganda are Jinja Regional Referral Hospital (Jinja) and Kitgum General Hospital (Kitgum), while in Malawi, the sites are Queen Elizabeth Central Hospital (Blantyre) and Kamuzu Central Hospital (Lilongwe). The two countries have similar guidelines and high parasite resistance rates to SP [18]. Jinja Regional Referral Hospital in east-central Uganda is a 250 referral centre on the shores of Lake Victoria. Kitgum General Hospital is a 100-bed district hospital in northern Uganda. Both hospitals serve areas with very high malaria transmission. The SCA clinic in Jinja runs weekly attending to about 80 outpatients. The one in Kitgum hospital is less developed and will be built further by study onset. In Malawi, the 1000-bed Queen Elizabeth Central Hospital is the largest tertiary hospital in the country. It has a long-standing paediatric SCA clinic serving over 400 children in southern Malawi. The dedicated SCA clinic in Kamuzu Central hospital was created in 2015 and, by the time of study onset, had over 200 patients on hydroxyurea treatment. In all four clinics, patients attend outpatient follow-up care every 1–3 months, and the guidelines for care are similar.

### Eligibility criteria {10}

#### Inclusion criteria

The inclusion criteria for enrolment are:

1. Children with a diagnosis of SCA *(HbSS)* on haemoglobin electrophoresis, high-performance liquid chromatography (HPLC) or isoelectric focusing
2. Aged between 6 months and 15 years
3. Weight of 5 kg or higher
4. Written informed consent from a parent or legal guardian and assent by the child (if aged 8 years or older)

The upper age limit of 15 years was chosen based on both a pragmatic approach of being able to recruit most patients attending SCA clinics and the lower incidence of malaria in adolescents beyond this age. Around Jinja, the incidence of malaria in children with normal haemoglobin (HbAA) declines by over 50% in adolescents 15–18 years compared to those 11–15 years (Dr Arthur Mpimbaza, unpublished).

#### Exclusion criteria

Participants presenting with an acute illness on enrolment will first receive treatment for the acute event and be recruited when symptoms resolve.

The exclusion criteria were:

1. Those with an additional chronic disease such as known congenital heart disease
2. Those with a known allergy to DP or SP
3. Other known red cell disorders, e.g. thalassaemia, G6PD deficiency
4. Those receiving daily cotrimoxazole prophylaxis
5. Those unlikely to comply with the follow-up schedule
6. Those participating in another trial

### Who will take informed consent? {26a}

Parents/guardians of children who fulfil the screening eligibility criteria will be approached and invited to the study clinic to provide written consent. Children 8 years or older will also be asked for assent. The consent (and assent where appropriate) will be obtained by the study nurse. The consent document will be provided in English or a local language understood by the parent/carer and participant.

### Additional consent provisions for collection and use of participant data and biological specimens {26b}

Separate consent will be sought for genetic testing, ECG and pharmacokinetic testing, for long-term storage and shipping of samples to external labs and for future research that may have no immediate clinical relevance.

### Interventions

#### Explanation for the choice of comparators {6b}

##### The rationale for the choice of DP for chemoprevention

SP is currently the anti-malarial drug of choice for malaria chemoprevention in SCA in most of sub-Saharan Africa. However, there is high-grade resistance to SP in many parts of the continent and Asia. Because of the long half-life of piperaquine (median 14 [range 10–18] days in children) [19, 20], DP provides 1 to 2 weeks longer post-treatment prophylaxis than other ACTs such as artemether-lumefantrine or amodiaquine-artesunate [21]. Amodiaquine has been evaluated (alone or combined with SP) for IPTp in Ghana. However, one of the amodiaquine arms was stopped prematurely because the drug was not well tolerated. Mefloquine provides a similar longer duration of prophylaxis compared to piperaquine, but mefloquine too is not well tolerated.

##### The rationale for once-weekly prophylaxis instead of monthly DP

Three pharmacological modelling groups have shown that in pregnant women, adults, and children, compared with monthly dosing, once-weekly DP would potentially lead to substantially higher steady-state piperaquine trough concentrations and result in a higher proportion of individuals attaining trough concentrations above the minimum inhibitory concentrations, while at the same time lowering the peak concentrations and the associated risk of QT prolongation [22–25]. Weekly DP is therefore predicted to be more effective than monthly dosing at preventing new infections and also exerting less selection pressure for piperaquine resistance. Importantly, these models also suggested that weekly dosing is more favourable than monthly administration in terms of missing occasional doses [23]. Moreover, although all three groups showed that the use of a loading dose with the standard 3-day regimen will reduce the time to reach steady-state concentrations, even without a loading dose, this can still be reached within 1 month [22–25]. Daily prophylaxis with low doses of DP was predicted to be even more favourable in terms of skipping doses than weekly or monthly regimens but was not considered an option for our trial because of the expected high pill burden in addition to others such as daily penicillin V, folic acid, and hydroxyurea. Daily or weekly prophylaxis with piperaquine monotherapy (i.e. without the DHA component) was not considered for this trial as (1) it is not currently available and (2) it would mean introducing monotherapy with piperaquine onto the market with the associated risk of development of parasite resistance.

#### Intervention description {11a}

Participants will be randomly allocated to receive one of two medications: weekly DP + monthly SP placebo or monthly SP + weekly DP placebo.

##### Dihydroartemisinin piperaquine (DP)

The study will use the scored dispersible tablets of D-ARTEPP® from Guilin Pharmaceutical Co. Ltd. D-ARTEPP is a WHO pre-qualified ACT. It is registered in both Uganda and Malawi. It will be provided as the co-formulated DHA (20 mg) and piperaquine (160 mg) and administered once weekly at approximate doses of DHA 2.5 mg/kg/day and piperaquine 20 mg/kg/day based on participants’ weight categories.

##### Sulphadoxine-pyrimethamine (SP)

The active control will be SP, the current standard of care for malaria chemoprevention for SCA in Uganda and Malawi. This will also be provided by Guilin Pharmaceutical Co. Ltd as their generic WHO-approved 500/25 mg tablets. It will be administered as monthly single-day courses of SP at approximate doses of S = 25 mg/kg and P = 1.25 mg/kg.

##### Placebo

Participants receiving weekly DP will in addition receive a single monthly dose of tablets identical to SP (SP-placebo). Those randomised to receive monthly SP will in addition receive weekly doses of tablets identical to DP (DP-placebo). In both trial arms, the dose and administration procedures will be identical to that for the active drugs.

#### Criteria for discontinuing or modifying allocated interventions {11b}

Participants will be withdrawn from study interventions for suspected or confirmed allergic reactions to the study drugs or for safety reasons as judged by the investigator, study safety monitor or the data safety and monitoring board (DSMB). Participants diagnosed with symptomatic uncomplicated malaria anytime during the study (unscheduled visits) will be treated with artemether-lumefantrine and where appropriate the next weekly dose of DP or the monthly dose of SP delayed.

#### Strategies to improve adherence to interventions {11c}

Right from the outset, participants and their parents/carers will be counselled on adherence to study medications. Adherence monitoring will be checked by a count of the remaining medication at every study visit. At all study visits, in addition to safety monitoring, residual pill counts will be performed to assess adherence and any challenges to that schedule. Subjects with more than a few residual tablets will be identified as less adherent to study requirements. Counselling will focus on the importance of adherence, and we will work with parents to troubleshoot issues. Parents will also be reminded not to share the study drug with siblings or other family members.

#### Relevant concomitant care permitted or prohibited during the trial {11d}

##### Routine care, outpatient care and permitted concomitant medication

Study participants will receive the current standard of care. All care directly related to the proper and safe conduct of the trial, and the treatment of immediate adverse events related to trial procedures will be provided free of charge by the study in the study hospitals. The provision of ancillary care beyond that immediately required for the conduct of the trial will not be covered by the trial. Thus, in addition to the study drugs:

- Parents/carers will be educated on the care of children with SCA. This will include sessions on daily activities including play and hydration, care of the child on hot and cold days, pain management, and when to seek medical help
- All will be supplied with a course of paracetamol for pain events
- Participants will also receive standard-of-care therapy and prophylaxis including:
  - ◦ Daily folic acid
  - ◦ Oral penicillin for children < 5 years
  - ◦ The immunisation of the child will be updated
  - ◦ Participants will be requested to attend the study clinic every 2 months

Because of concerns about drug-drug interactions between SP and folic acid and the potential impact on malaria chemoprophylaxis [26], folic acid supplementation will be provided at the internationally recommended dose of 0.4 mg daily.

##### Hospitalisation and inpatient care

The following will necessitate hospitalisation and inpatient care:

1. Development of severe complications including severe anaemia (Hb < 5 g/dl), severe pain episodes/vaso-occlusive crises, stroke, acute chest syndrome, mesenteric crisis, hyper haemolytic crisis and a hepatic crisis among others
2. In addition to a blood slide for malaria parasites, participants presenting with axillary temperature > 38.0 °C will have blood and urine cultures taken and be given iv antibiotics (ceftriaxone). Further management will depend on blood slide, fever response and culture results. An episode of malaria will be defined as fever plus the presence of *P. falciparum* on a blood smear. Severe malarial anaemia will be defined as the presence of *P. falciparum* on blood smear and haemoglobin ≤ 5 g/dL. During the treatment and follow-up phase of the study, if a subject is diagnosed with malaria, the investigator will prescribe antimalarial treatment based on the severity of the malaria illness. The World Health Organization criteria will be used to classify children as having severe or uncomplicated malaria
3. Need for surgical procedures

Hospitalised participants will be managed according to local standard guidelines. The standard in-hospital care for severe anaemia includes a blood transfusion (20 ml/kg whole blood or 10 ml/kg packed red cells). If this is due to malaria, the child will in addition receive parenteral artesunate (2.4 mg/kg at times 0, 12, and 24 h [children weighing < 20 kg will receive a higher dose of artesunate—3 mg/kg per dose]) and thereafter once the patient can take orally, a full 3-day course of artemether-lumefantrine.

##### Prohibited medication

Prohibited medication includes antimalarial drugs not prescribed within the trial protocol and other drugs with antimalarial properties such as co-trimoxazole. Therefore, concomitant use of the antimalarials mefloquine, halofantrine, tetracyclines, artemisinin derivatives, sulphadoxine/pyrimethamine, atovaquone-proguanil (Malarone) and chlorproguanil-dapsone (Lapdap™) are not allowed from baseline until end of the study. Randomised participants who take prohibited medications will remain in the trial and will be included in the primary, intention-to-treat analysis, but excluded from the per-protocol analysis.

##### Concomitant therapy

All concomitant medications taken during the study will be documented with the indication, dose information and dates of administration.

#### Provisions for post-trial care {30}

At the end of the intervention, unless policies change, participants will revert to the standard of care treatment. The study budget is not in a position to fund post-study care or implementation of weekly DP as policy. However, the investigators will be working in close collaboration with local (Ministry of Health) and international policymakers (e.g. WHO) to ensure that policymakers are informed early of any pertinent research findings.

### Outcomes {12}

#### Primary efficacy outcome

The primary efficacy outcome is the incidence of clinical malaria defined as a history of fever in the preceding 48 h or documented axillary temperature of ≥ 37.5 °C, plus *P. falciparum* parasites on microscopic examination of a blood smear by a certified malaria microscopist.

#### Key secondary outcomes

1. Clinic visits because of blood smears or malaria rapid diagnostic tests (RDT) confirmed non-severe and severe malaria
2. All-cause sick-child clinic visits after enrolment
3. Incidence of malaria parasitaemia
4. All-cause hospital admissions after enrolment
5. All-cause and malaria-specific hospitalisation
6. SCA-related vaso-occlusive events (including severe pain events and dactylitis), acute chest syndrome, stroke and need for blood transfusion
7. All-cause mortality after enrolment

#### Other outcomes

##### Safety outcomes

1. Incidence of serious adverse events (SAEs; grade 3 or 4 adverse events) according to the Common Terminology Criteria for Adverse Events (CTCAE) toxicity tables and coded using MedDRA
2. Adverse events (grade 1 or 2 toxicity) after enrolment
3. Incidence of serious cardiac adverse events (convulsions or syncope within 48 h after drug intake)
4. Change in QTc length and QTc-prolongation on four-monthly ECG recordings and by the age-dependent cut-off value (in a subset of patients)

##### Tolerance

1. Vomiting the study drug within 30 min of administration of the study medication
2. Incidence of gastrointestinal complaints after enrolment

##### Operational feasibility, acceptability, uptake and compliance

1. User and provider experiences of the intervention
2. User satisfaction with the intervention
3. User treatment preference
4. Adherence

##### Cost-effectiveness

1. Provider costs of delivering the interventions and provider costs of managing malaria in SCA children
2. Direct and indirect costs of patients receiving the interventions and managing cases of malaria
3. Incremental cost-effectiveness of replacing current standards of care (SP) with DP from the perspectives of the health care provider and the society
4. Health-related quality of life

##### Genetic markers of resistance

1. Development of markers of resistance to piperaquine such as plasmepsins 2 and 3
2. Range of antimicrobial resistance in bacterial isolates on blood and urine culture in febrile children

### Participant timeline {13}

**Fig. 1:**
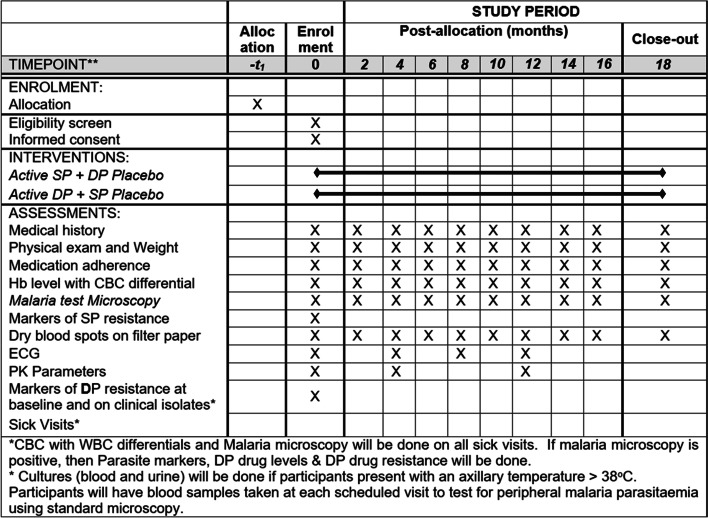
Showing study flow chart

Participant timeline is presented in Fig. 1.

### Sample size {14}

The minimum incidence rate of clinical malaria in SCA patients receiving monthly SP is estimated at 0.2 events per year. Assuming an effect size of 50% if DP is used (i.e. a 50% reduction in the rate as in Bigira et al. [27] that is, a hypothesised incidence rate of 0.1 events per person per year in the DP group), at a power of 0.9 and 0.05 level of significance, we will need 438 individuals to be followed up for 18 months. Allowing for 20% losses to follow-up, we shall need 548 participants (274 in each group) to be followed for an average of 18 months, equivalent to 9864 person months, or 824 participants for an average follow-up of 12 months, equivalent to 9888 person-months.

### Recruitment {15}

Recruitment will be done simultaneously across all sites and will be competitive between the sites in the trial.

## Assignment of interventions: allocation

### Sequence generation {16a}

An independent statistician will generate the randomisation sequence in randomly varying block sizes stratified by site and weight band and keep the randomisation code in a locked computer file.

### Concealment mechanism {16b}

Allocation concealment is achieved by having patients randomised to DP also receive an SP placebo, and those on SP will, in addition, receive a DP placebo.

### Implementation {16c}

The randomisation list and the dummy codes generated by the independent statistician and the allocation sequence will be forwarded to the trial pharmacists in Uganda and Malawi to prepare sequentially numbered, sealed envelopes for the participants. The pharmacists will prepare the subject-specific study drugs by site and weight bands following the randomisation scheme. Participants will be enrolled competitively, assigned a registration number in sequential order, and treatment arm by trial staff.

## Assignment of interventions: blinding

### Who will be blinded {17a}

All study staff will be blinded to the treatment assignment, including the investigators, outcome assessors and trial statisticians. In addition, both the participants and caregivers will also remain blind to the intervention arms assigned to individual patients. The randomisation code will be kept in a locked computer file with the independent statistician and made available to the chair of the DSMB on request.

### Procedure for unblinding if needed {17b}

Emergency unblinding will be on an individual basis to ensure that the blinding of other participants is not affected. It will be considered if a suspected unexpected serious adverse reaction to the study drug (SUSAR), serious adverse reaction to the study drug or serious expected adverse drug-drug interactions between the study drug and other drugs provided to the participant undergoing treatment, as judged by the study physician or study safety monitor. The first alert will be raised by the study physician within 24 h of becoming aware of the event in an expedited report to the chief investigator, ethics committees, safety monitor, sponsor and DSMB chair. The final decision will be advised by the country’s principal investigator and/or the chief investigator in consultation with the safety monitor and/or DSMB chair. If clinically indicated, the subject will be withdrawn from receiving further study drugs. Other than the written or verbal disclosure of the code in any of the confidential correspondence about the participant between the principal or chief investigator and the safety monitor or the DMSB chair, the actual allocation will NOT be disclosed to the participant and/or other study personnel including other site personnel, monitors, corporate sponsors or project office staff.

Unblinding of data in nested studies such as the pharmacokinetic study will be done by independent statisticians not involved in the main trial to ensure the blinding of other participants is not affected. A database with the results of the nested study and the corresponding data from the main trial for these participants will be emailed to the trial statistician. The trial statistician will pseudonymise the data, such that the personal data can no longer be attributed to a specific study subject without the use of additional information.

## Data collection and management

### Plans for assessment and collection of outcomes {18a}

Before study onset, all the assessors (clinical, nursing, laboratory, pharmacy and data) will undergo study-specific training on study procedures. The internal and external monitors will independently document readiness in the site initiation visits before recruiting the first participant. Twice annual refresher training will be carried out and especially following the subsequent interim monitoring visits.

Following the enrolment visit, participants will return for follow-up at 2 months and then every 2 months thereafter until the required person-months of observation is achieved. The study will refund the costs of transport to the clinic and provide a time compensation. At each visit, all clinical events in the intervening period will be ascertained and those previously not reported or for which the patients did not attend the study clinic will be documented. Participants with < 80% adherence at any one point will have additional counselling.

To determine the outcomes, any participant with a history of fever in the previous 48 h or temperature ≥ 37.5 °C on any visit will have a blood smear for malaria microscopy performed by a certified malaria microscopist. All other SCA-related and non-SCA-related events such as painful crises and hospitalisations will also be documented and reported. In this way, the point prevalence at each visit and the incidence of malaria can be estimated for each study arm without contaminating the intervention in either arm.

The study will provide a standard of care clinical services for sick visits and refer for appropriate care if the level of care required is beyond the capacity of the clinic. All these episodes will be reported as adverse events according to severity. All the adverse events (AEs) and serious adverse events (SAEs) during the intervening period will be documented following ICH GCP principles and all SAEs reported to regulatory bodies, sponsors, DSMB and ethics committees.

### Plans to promote participant retention and complete follow-up {18b}

To minimise losses to follow-up and be able to follow-up participants, contact mobile cell phone numbers will be recorded at recruitment and a sketch map of the home location of the study participant drawn. A study home visitor will document the route home together with Global Positioning Satellite (GPS) data of the home. In the event the participant does not attend the scheduled follow-up visits, this information will be used for tracing.

### Data management {19}

#### Data collection

Data will be collected and recorded at the point of contact by one of the trained study staff. The study will use a combination of paper-based (baseline and study end) and electronic record forms. Barring logistical difficulties, all intervening nursing and clinical data will be entered directly into password-protected databases. We will use the Research Electronic Data Capture (REDCap™), a secure, web-based international data capture system. This will be hosted at servers at Global Health Uganda and the Training and Research Unit of Excellence in Malawi.

#### Data sharing

At the end of the study, anonymised data will be available to other researchers for further analysis, subject to ethical approval, the terms of the original patient consent and agreement on its use according to prevailing laws on intellectual property rights.

#### Archiving

Hard copies of CRFs will be stored long-term at secure storage facilities for a minimum of 5 years. Data will also be kept electronically in a public data repository in compliance with prevailing laws on data storage.

#### Data quality and standards

The quality of the electronic and questionnaire data collection and data entry will be maximised through the training of field staff in standardised questionnaire administration, in the methodology for collecting data and all staff will be expected to demonstrate competence before conducting fieldwork. All electronic CRFs and data validation processes for data will incorporate range and consistency checks.

#### Managing, storing and curating data

Verified and validated data will be stored via the cloud on a secure server. The investigators will have access to the site data on the central server. Locally, data will be backed-up continuously on a secure off-site server and/or encrypted standalone hard drives. Once the data validation phase is completed by the central data manager, the database will be locked and transferred to a statistical programmer who will do further syntax-driven consistency checks and syntax-driven data cleaning (e.g. in Stata). Once data entry and cleaning are complete, hard copies of CRFs will be stored long-term at secure storage facilities in Malawi and Uganda as per local storage policies and guidelines. Data will also be kept electronically in compliance with prevailing laws on data storage. The research data will be stored for the long term in the original electronic format, in a large unified database and a public database that contains all research data other than participant identifiable data.

### Confidentiality {27}

All information regarding the participants will remain confidential to the extent allowed by law. Unique numerical identifiers will be used for data entry. All screening forms and case report forms will be kept in a secured location with access limited to authorised study staff. Unique numerical identifiers will be used for the computer-based data entry and blood samples. Publications will contain only aggregated data. No identifying information will be included to ensure individual patient anonymisation of all data and results made public.

### Plans for collection, laboratory evaluation and storage of biological specimens for genetic or molecular analysis in this trial/future use {33}

A 2-ml sample of blood will be drawn on enrollment for a blood smear for malaria parasitaemia using standard microscopy, complete blood count and for a dry spot on filter paper. This will be repeated during the scheduled and unscheduled visits at which time if clinically indicated, additional samples will be drawn for culture (blood and urine) from participants with axillary temperatures > 38 °C. The filter paper samples will be used to classify parasitaemia as recrudescence or re-infections by comparing alleles of parasite genes encoding merozoite surface proteins 1 and 2 (*msp1*, *msp2*) and glutamate-rich protein (*glurp*) [28] using standard PCR methods and determining resistance to SP and piperaquine [29]. The antimicrobial susceptibility and resistance patterns of bacterial isolates in febrile children will be examined to determine the impact of malaria prophylaxis on antimicrobial drug resistance. The remaining cell pellet, plasma and serum samples will be stored and in future, used for immunological (e.g. antibodies against viral infections) and the role of sickle cell variants (genetic).

## Statistical methods

### Statistical methods for primary and secondary outcomes {20a}

#### Analysis populations

Primary analyses will be based on intention-to-treat (ITT) population, and secondary supportive analyses will be done on the per-protocol (PP) population, which is important for cost-effectiveness considerations. Incidence rates will be calculated and rate ratios estimated using Poisson regression, with treatment (as randomised) as the only predictor variable and the stratification factors of site and weight band as co-variates.

1. Intention-to-treat (ITT) population: This population consists of all randomised subjects who have a valid outcome
2. Per-protocol (PP) population: This population is a subset of the ITT population. Subjects with major protocol deviations will be excluded from the PP population. Major protocol deviations will be defined in the SAP

#### Assessment of efficacy

We will compare incidence rates between intervention groups using incidence rate ratios. Incidence rates will be calculated as the number of events divided by the total follow-up time in each arm. Rate ratios shall be estimated using Poisson regression, with treatment (as randomised) as the only predictor variable and the stratification factors of site and weight band as covariates. In the presence of overdispersion, negative-binomial regression will be used to estimate the incidence rate ratios. Patients who die, withdraw or get lost in follow-up will be censored at their date of death, withdrawal or the last follow-up visit. A *P*-value < 0.05 will be considered statistically significant.

#### Secondary efficacy outcomes

Intention-to-treat analysis shall be followed for analysis of secondary outcomes. For count secondary outcomes, all sick visits, malaria-specific sick visits, all-cause hospitalisations, malaria-specific hospitalisations, serious cardiac adverse events and SCA-related vaso-occlusive events, we shall fit Poisson regression models and compare events between the intervention groups using incidence rate ratios. Negative-binomial regression models will be fitted if there is overdispersion. Model adjusters shall be informed by imbalances in baseline characteristics between the intervention groups. We shall fit a binary logistic model to compare the odds of death between the two intervention groups. Death shall likely be a rare outcome in this study, and often, binary logistic regression models face convergence problems due to the sparse outcome. We shall instead fit models using the firth method, which avoids the convergence challenges of the logistic regression model when the outcome is rare. We shall report odds ratios as measures of association. We shall also test if the change in QTc length from baseline is > 60 ms using a one-sided *t*-test. We will provide a list of individual participant changes in QTc length from baseline. Exploratory quantitative outcomes shall be summarised by the intervention arm using descriptive statistics. Qualitative exploratory outcomes such as user and provider experiences of the intervention, satisfaction with the intervention and treatment preference will be described as text. We shall also present a list of adverse events and document if they were related to the study drug or not.

### Interim analyses {21b}

Only one interim efficacy analysis is planned to assess the primary outcome when the study reaches approximately 50% follow-up time, i.e. about 412 person-years follow-up per study arm time accumulated across all study sites or at least half of the estimated number of events (62 out of an anticipated 124 events across both arms). This information will be used to determine if the study should continue as planned, proceed with modifications or be terminated. The Haybittle–Peto boundary will be used as a stopping rule, which states that a trial could be stopped early if an interim analysis shows a probability ≤ 0.001 that a difference as extreme or more between the treatments is found, given that the null hypothesis is true.

### Methods for additional analyses (e.g. subgroup analyses) {20b}

#### Subgroup analyses

For sub-analysis, we will use stratified analysis to assess to what extent the effect of the intervention on the primary outcome is influenced by site, demographic (e.g. age, and socio-economic status) or clinical parameters, malaria transmission variables (malaria transmission intensity, residence (urban/rural), season, insecticide-treated nets use, site), time of assessment and potential intervention modifiers.

#### Sensitivity analyses

Several sensitivity analyses will be conducted to assess the robustness of the primary endpoint analysis. These include an analysis of the PP subject population and a covariate-adjusted analysis. Other regression models will also be explored. Additional post hoc analyses may also be conducted if deemed appropriate. In addition, we will compare the results of the covariate-adjusted analyses with and without imputation for missing values for covariate values at baseline.

### Methods in analysis to handle protocol non-adherence and any statistical methods to handle missing data {20c}

Primary analyses will be ITT analyses, which will include all participants as randomised regardless of their adherence to the intervention. Every effort will be made to minimise the amount of missing data in the trial, and whenever possible, information on the reason for missing data is obtained. No adjustments will be made for missing outcome data, but missing data may be imputed for covariates.

### Plans to give access to the full protocol, participant-level data and statistical code {31c}

The full protocol will be available on request to any interested professional and be published in peer-reviewed journals and deposited in an online repository. Individual, de-identified participant data will be made available for meta-analyses as soon as the data analysis is completed, with the understanding that the results of the meta-analysis will not be published before the results of the individual trial or without the prior agreement of the investigators. A fully de-identified data set of the complete patient-level data will be available for sharing purposes, such as via the WWARN repository platform (http://www.wwarn.org/working-together/sharing-data/accessing-data) no later than 5 years after the publication of the trial. All requests for data for secondary analysis will be considered by the Data Access Committee to ensure that the use of data is within the terms of consent and ethics approved.

## Oversight and monitoring

### Composition of the coordinating centre and trial steering committee {5d}

A project management committee will be comprised of the principal investigators, all the co-investigators and trial coordinators/managers in both countries. It will meet monthly by teleconference and in person in years 1, 3 and 5 to review progress and make strategic decisions. However, no trial steering committee was named as it was felt the DSMB will cover this role.

### Composition of the data monitoring committee, its role and reporting structure {21a}

The DSMB will consist of 3–5 members (including senior clinicians from Uganda and Malawi, and a senior statistician). The chair will be a clinician with substantial clinical trial experience. DSMB members shall be the only persons to look at the safety results, to prevent participants from being exposed to any excess risks by recommending trial suspension or termination if there are convincing safety concerns. The chief investigator and trial statistician shall attend part of the DSMB meeting to present the most current data. This will be blinded unless the DSMB specifically requests an unblinded analysis.

### Adverse event reporting and harms {22}

All AEs and SAEs will be documented following ICH GCP principles and all SAEs reported to the regulatory bodies, sponsor, DSMB and ethics committees using an SAE form electronically. This will include the nature of the event, date of onset, severity, corrective therapies given, outcome and causality (i.e. unrelated, unlikely, possible, probably, definitely). All SAEs will be reported to the local ethics committee and DSMB within seven [7] days of the investigators becoming aware of the event. Thereafter, additional information or a detailed report of the SAE should be submitted within another [7] days.

### Frequency and plans for auditing trial conduct {23}

Four internal and external monitoring visits each are planned. In addition, representatives of the sponsor, ethics and regulatory authorities may visit the study sites and perform audits or inspections, including source data verification. These bodies will also have direct access to source data and other relevant documents as required.

### Plans for communicating important protocol amendments to relevant parties (e.g. trial participants, ethical committees) {25}

If the protocol must be amended, any amendments will be submitted to the research ethics committees at LSTM (sponsor) and the primary ethics committees in Uganda and Malawi for approval before implementation, as well as to the funder, the Norwegian Research Council. Any change to the informed consent form, except for changes in layout, spelling errors and formatting, must also be approved by the sponsor and the primary ethics committee before the revised form is used. No change will be made to the approved protocol without the agreement of the sponsor.

## Dissemination plans {31a}

The results of the study will be published and disseminated at national and international conferences and in peer-reviewed scientific journals. A final report will also be distributed to all local collaborating partners, district and provincial and national health authorities in combination with a local workshop to be held at country offices and to the Norwegian Research Council.

## Discussion

If proven safe and efficacious, chemoprevention with DP may decrease the incidence of malaria in patients with SCA, prevent ill health and deaths and improve well-being. To this effect and even before the results are out, we have initiated dialogue with the National Malaria Control Programs at the Ministries of Health in East, Central and Southern Africa from the outset and the WHO Global Malaria Program (GMP) on policy decisions and the potential for scale-up to further optimise our prior engagements from previous and ongoing trials. We conducted stakeholder mapping and constituted a working group with representatives of the above key actors, the investigators and representatives of consumer/patient advocacy groups. This stakeholder platform did input into the study protocol at a 2-day inception meeting during protocol development. Later, a second policy dialogue will deliberate on study results and develop a draft policy on implementation and scale-up. In between, we will present progress reports to the different bodies. In the end, we shall develop policy briefs using the graded entry approach [30]. This will include discussion papers on results, safety and risk of drug resistance, acceptability and barriers to uptake and cost-effectiveness, a systematic review and a meta-analysis to update the existing literature on Chemo-prevention of Malaria in Children with Sickle Cell Anaemia.

### Trial status

This manuscript is prepared from protocol version 2.1 dated 1 January 2022. Participant recruitment commenced on 30 April 2021 and follow-up is ongoing.

## Data Availability

The full study protocol, supporting documents and the fully anonymised research database will be made publicly available once the study findings have been published. The data manager and statistical programmer will produce a document summarising the methods used to generate the data with a full description of all procedures, analyses, data capture tools, coding and description of variables. This document will be available alongside the research database.

## Abbreviations

ACT: Artemisinin-based combination therapy
AE: Adverse event
AIDS: Acquired immunodeficiency syndrome
CHEMCHA: Chemoprevention of malaria in children with sickle cell anaemia
CRF: Case report form
CRO: Contract Research Organisation
ECG: Electrocardiogram
DHA: Dihydroartemisinin
DHFR: Dihydrofolate reductase
DHPS: Dehydropteroate synthetase
DP: Dihydroartemisinin-piperaquine
DSMB: Data safety and monitoring board
G6PD: Glucose-6-phosphate dehydrogenase
GCP: Good Clinical Practice
LSTM: Liverpool School of Tropical Medicine
HIV: Human Immuno-deficiency virus
IEC/IRB: Independent Ethics Committee/Institutional Review Board
ITT: Intention to treat
ICH: The International Conference on Harmonisation
IPTpd: Intermittent Preventive Therapy post-discharge
PCR: Polymerase chain reaction
PK: Pharmacokinetic
SD: Standard deviation
SOP: Standard operating procedure
SP: Sulphadoxine-pyrimethamine
SSA: Sub-Saharan Africa
T1/2: Plasma half-life
Tmax: Time to maximum plasma concentration
URTI: Upper respiratory tract infection
QRS: The time interval between Q-, R- and S- waves on ECG records
QT: The time interval between Q to T-wave (interval of electrocardiogram)
QTc: QT corrected
RBC: Red blood cells
SAE: Serious adverse event
WHO: World Health Organization
